# Evaluation of the decision-making process within the table-top exercise of the Terror and Disaster Surgical Care (TDSC^®^) course

**DOI:** 10.1007/s00068-022-02217-1

**Published:** 2023-02-15

**Authors:** Gerhard Achatz, Alexander Reckziegel, Benedikt Friemert, Markus Blätzinger, Simone Hinz-Bauer, Axel Franke, Dan Bieler, Thomas Paffrath, Patrick Hoth, the Deployment, Disaster, Tactical Surgery Section of the German Trauma Society

**Affiliations:** 1 Department for Trauma Surgery and Orthopaedics, Reconstructive and Septic Surgery, Sportstraumatology / Trauma Research Group, German Armed Forces Hospital Ulm, Oberer Eselsberg 40, 89081 Ulm, Germany; 2 AUC-Academy for Trauma Surgery GmbH, Munich, Wilhelm-Hale-Straße 46B, 80639 Munich, Germany; 3 Department for Trauma Surgery and Orthopaedics, Reconstructive and Hand Surgery, Burn Medicine, German Armed Forces Central Hospital Koblenz, Rübenacher Straße 170, 56072 Koblenz, Germany; 4 Department of Orthopaedics and Trauma Surgery, Heinrich Heine University Hospital Düsseldorf, Moorenstraße 5, 40225 Düsseldorf, Germany; 5 Department for Trauma Surgery, Krankenhaus der Augustinerinnen, Jacobstr. 27-31, 50678 Cologne, Germany

**Keywords:** Terror, Disaster, Preparedness, Decision-making, Table-top exercise, Simulation game, Evaluation

## Abstract

**Purpose:**

The threat of terror is omnipresent in Europe and the number of attacks worldwide is increasing. The target of attacks in Europe is usually the civilian population. Incalculable dangerous situations at the scene of the event and severe injury patterns such as complex gunshot and explosion injuries with a high number of highly life-threatening people present rescue forces, emergency physicians and subsequently hospitals with medical, organizational as well as tactical and strategic challenges. The Terror and Disaster Surgical Care (TDSC^®^) course trains clinical decision-makers to meet these challenges of a TerrorMASCAL in the first 24–48 h.

**Methods:**

A table-top exercise was developed for the TDSC^®^ course as a decision training tool, which was prospectively evaluated in six courses. The evaluation took place in 3 courses of the version 1.0, in 3 courses in the further developed version 2.0 to different target values like, e.g., the accuracy of the in-hospital triage. Furthermore, 16 TDSC^®^ course instructors were evaluated.

**Results:**

For the evaluation, *n* = 360 patient charts for version 1.0 and *n* = 369 for version 2.0 could be evaluated. Overall, the table-top exercise was found to be suitable for training of internal clinical decision makers. Version 2.0 was also able to depict the action and decision-making paths in a stable and valid manner compared to the previous version 1.0. The evaluation of the instructors also confirmed the further value and improvement of version 2.0.

**Conclusion:**

With this prospective study, the table-top exercise of the TDSC^®^ course was tested for decision stability and consistency of the participants’ decision paths. This could be proven for the selected target variables, it further showed an improvement of the training situation. A further development of the table-top exercise, in particular also using digital modules, will allow a further optimization.

http://www.bundeswehrkrankenhaus-ulm.de

## Introduction

### Background

The terrorist threat in Europe is omnipresent, and the number of terrorist-motivated attacks worldwide is increasing [1]. To prepare and train medical staff for the specifics of a terror-associated mass casualty incident (TerrorMASCAL), the Terror and Disaster Surgical Care (TDSC^®^) course was developed in 2017 by the German Trauma Society (Deutsche Gesellschaft für Unfallchirurgie/DGU) in collaboration with the Deployment, Tactical and Disaster Surgical Section of the DGU. By the time data collection for this paper began in 2019, 465 participants had already been trained in previous courses. The two-and-a-half-day TDSC^®^ course provides important content on the characteristics of a TerrorMASCAL, associated injury patterns, and strategic and tactical solutions for dealing with TerrorMASCAL scenarios in the hospital setting. For practical implementation of the course content, there was a table-top exercise developed specifically for the TDSC^®^ course. This decision-making training is a progressively simulated group scenario that guides participants to develop and understand the implications of a potential mass casualty incident given limited capacity and to learn resolution algorithms.

Within the exercise, the objective is to treat as many of the injured as possible under the given conditions of an appropriate scenario using special patient cards.

To achieve this, the medical treatment must be controlled in the best possible way, making optimum use of the available resources. In principle, treatment can be carried out using three different concepts: early total care (ETC), damage control surgery (DCS) or tactical abbreviated surgical care (TASC).

The participants should directly incorporate the theoretical content learned in the course on medical as well as strategic and tactical characteristics of TerrorMASCAL -situations into their decisions.

Thus, the table-top exercise of the TDSC^®^ course uses the approach of playful knowledge transfer to ensure the interest, motivation and learning progress of the target group, namely experienced clinical decision-makers. In the literature, there are only a few evaluated board games in the field of medical education. An evaluation of the decision training/table-top exercise as a board game in the context of the TDSC^®^ course seemed obvious and the following hypothesis should be answered:

With the Table-Top Exercise of the TDSC^®^ course, further terror preparedness of in-hospital decision-makers is possible. Furthermore, the table-top exercise can be used in the context of the further development from version 1.0 to version 2.0 validly and without loss of quality.

### Training status and need for physicians for a TerrorMASCAL

Special training for clinically active physicians and especially surgeons to prepare for attacks and quality-assured treatment of terror victims is needed, according to the clear result of a further assessment and evaluation by the German Trauma Society [2]. It has already been demonstrated in the past that medical triage, for example, is not communicated in a standardized manner and thus its reliability cannot be assessed without doubt [3]. However, since, for example, the initial in-hospital triage is a very central and necessary aspect for the management of such an event, since the utilization of the available resources depends significantly on it. This underlines the importance and necessity for training of in-hospital decision-makers and important key personnel, such as a Senior Triage Coordinator (LArS) as well as a Emergency Operational and Medical Coordinator (EOMC = ZONK).

Various training formats have already been established internationally, some of which use different didactic methods. The TDSC^®^ course chooses the approach of educational knowledge transfer, interactive case and scenario discussions as well as a practical knowledge transfer by means of a table-top exercise [10]. In this context, the value of the use of simulation models in medical education for interactive training of response to major incidents and disasters has already been increasingly described in recent years [4]. Furthermore, it can be seen that board games in particular have a positive influence on the actions of the person being trained [5]. In doing so, Skryabina et. al. presented in 2017 that exercises to prepare for health emergencies are effective and improve participants' knowledge of emergency activities, policies and procedures and overall competence and confidence [6]. The current Level 3 “guideline on the treatment of patients with severe/multiple injuries” also endorses the usefulness of simulation games [8]. This shows that appropriate preparation is the best basic prerequisite for facing an MASCAL despite all eventualities. In addition to the analysis of damage situations that have already occurred, the guideline specifies that simulation games are a suitable means for the further development of medical personnel in preparation for an MASCAL [7, 8].

## Methods

### Study group description for the table-top exercise

For the present prospective study, the table-top exercise was evaluated with 18 play groups and thus a total of 49 course participants in 3 TDSC^®^ courses with version 2.0. The courses took place after the resumption of classroom training after the COVID-19 pandemic in 2021. In advance, a similar analysis of version 1.0 of the table-top exercise had already taken place in 2019 before the pandemic; here, 19 game groups with 55 course participants were evaluated. In total, *n* = 360 patient cards for version 1.0 and *n* = 369 patient cards for version 2.0 could be considered and evaluated.

### Relevant elements of the table-top exercise

The table-top exercise was already presented by our working group in 2020 in the context of a publication in this journal and will therefore only be described in abbreviated form here [10].

The aim is for a group of 3 course participants to provide in-hospital care for the patients resulting from a mass casualty incident within the framework of a given scenario based on a hospital structure depicted on a game board. Within the group of 3, the following tasks are performed in terms of content: Senior Triage Coordinator (LArS), Emergency operational and medical coordinator (ZONK) as well as ZONK helper, the latter is assigned and essentially occupied with leading the whiteboard. A corresponding decision-making process can always take place in the team during the exercise, but the ZONK may have to make a final decision.

**Fig. 1:**
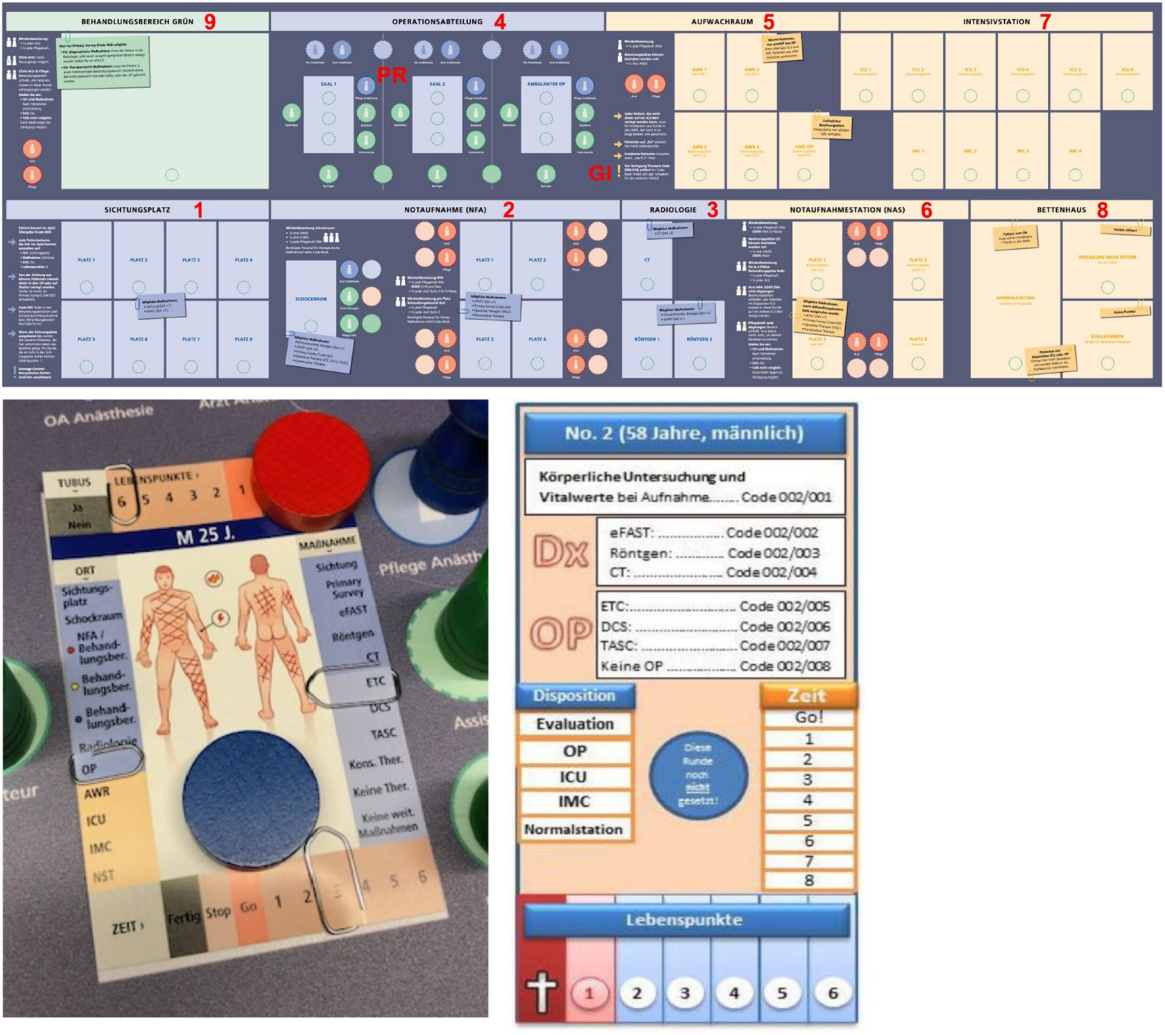
The game board from the table-top exercise is shown above. Number 1 represents the screening area, where patients are first screened and categorized. From here, patients are taken to the emergency area/treatment area red (number 2) with a connection to the radiology department (number 3) for further treatment prioritization after a primary survey and appropriate diagnostics, if necessary. In the further course, depending on the previous examination results and capacities, the patients are taken to the operating room (number 4) or to the intensive care unit (number 7). Following surgery, monitoring is required from the patient in the recovery room (number 5). Patients with mild injuries or patients not triaged in red can be cared for in the emergency department/treatment area yellow (number 6) or in the ward block (number 8). Patients triaged green are cared for in the treatment area green (number 9) [9]

After the initial triage, the patients are assigned to the corresponding triage areas of the triage categories red, yellow, and green (Categorizing). After a further evaluation of the patients in the sense of a primary survey (Prioritizing), the determination (Coordinating) of any necessary diagnostics, the realization of the corresponding care and finally the inpatient admission, e.g., to the intesive care unit (ICU), the intermediate care unit (IMC) or the normal ward (Implementing). The following Fig. 1 shows again schematically the board of the table-top exercises as well as the used patient cards of the versions 2.0 and 1.0.

The so-called patient cards of the new (left/version 2.0) and the old (right/version 1.0) table-top exercise are shown below [2]. Information is given about the wounded person. Furthermore, the following can be set on the cards and thus also read off: in addition to the information on the patient cards from version 1.0 with the information on age, gender, measures to be performed, treatment concept, time course and so-called life points as an expression of the physiological status, information on the ventilation status as well as a graphic injury representation in the sense of a pictogram with legend have been added to the patient cards of version 2.0 [10].

A codebook, in which the respective patient findings and histories as well as their further courses and, for example, examination results or also operation or treatment procedures are specified, guides the participants through the individual patient paths. Thus, the final course of the individual patient or even of the entire clinical process remains dependent to a large extent or only on the decision-making behavior of the participants.

The aim is to provide as many patients as possible with the best possible, but also the most sensible care, depending on resources. In the context of the overall situation, restrictions and compromises with regard to diagnostic options, further care and, above all, (surgical) therapy may have to be taken into account and accepted to ensure the survival of as many patients as possible. In these decision paths, which are, thus, also very resource dependent, it may be necessary to choose care options that are not known or conceivable from everyday clinical practice. For example, if an urgent surgical treatment of a limb injury to preserve the limb is postponed in favor of an operation to save the survival of another patient with a trunk injury and active bleeding, and thus the loss of the limb may have to be accepted. This would then correspond, for example, to a procedure in the sense of tactical abbreviated surgical care (TASC), which is not known to clinical physicians and also surgeons and is not required in normal daily business.

In the scenario presented and used for the evaluation, the clinical infrastructure of the table-top exercise was confronted with 20 patients each after a fictitious explosion scenario in the context of a rock concert with more than 2000 spectators. Thereby, the order of the patients' arrival and the existing order of injuries were always the same, provided and adjusted random events by so-called event cards as well as alerting cards (responsible for the addition of subsequently alerted personnel) for all examined game sequences were likewise the same. With these and even further corresponding adjustments, a completely identical and immediately unambiguous game situation could be created for the game rounds and their evaluation.

### Study implementation

Descriptive statistics were aggregated on the basis of six table-top exercises, comparing the evaluation of three courses with version 2.0 and evaluation of three courses with the older version 1.0.

A total of 729 patient charts could be evaluated. Data collection regarding the main outcome measure: hit accuracy of the sighting categories and the secondary outcome measures: location at the end of the game (allocation of patients to treatment areas), diagnostics and therapy measures was always performed in the second of a total of four exercises of a corresponding TDSC^®^ course under lecture hall conditions. During the 90-min table-top exercise, patient cards were played four times at a fixed time by a so-called game leader. In total, there were *n* = 20 patient cards for each group. Due to a lack of time with a fixed end of the game after 90 min, some of the following patients were not seen by the groups during the evaluation of the table-top exercise 1.0 and were, therefore, excluded from the game evaluation: patient 7 was not sighted by two groups, Patient 8 was not sighted by three groups, Patient 9 was not sighted by six groups. Consecutively, a total of 11 patient cards were excluded from the game evaluation in game 1.0.

### Instructor evaluation for further improvement of the table-top exercise

The evaluation of the course instructors always took place in the context of the evaluated courses direct after the above-mentioned table-top exercises of the version 2.0 in the year 2021. All instructors had already accompanied TDSC^®^ courses several times beforehand and were therefore also intensively familiar with version 1.0.

A self-designed, anonymous questionnaire with a total of 23 questions was used. The answer options to the 23 questions were divided into a nominal and ordinal scale, including the possibility of single answers. For three questions, there was an additional free expression of opinion on the answer option. The questionnaire was distributed to 16 instructors. The questionnaire investigated the following basic topics:

- instructors' level of experience?
- expected and realized improvement of the terror preparedness of the participants by the table-top exercise?
- optimization of the table-top exercise in version 2.0?
- explainability and presentation of version 2.0 compared to version 1.0?

A total of 16 (100%) of the questionnaires could be obtained for the evaluation.

Thereby 11 questions were considered for the present publication, which covered above listed main topics in content. The remaining 12 questions did not directly deal with the topic of the objective for this publication and were, therefore, not considered in the present publication, since no thematic context was given with it.

## Results

### Study group analysis

For version 1.0 of the table-top exercise, *n* = 55 participants (*n* = 6, 12% females) and for version 2.0 *n* = 49 (*n* = 12, 22% females) were included in the present study.

**Fig. 2:**
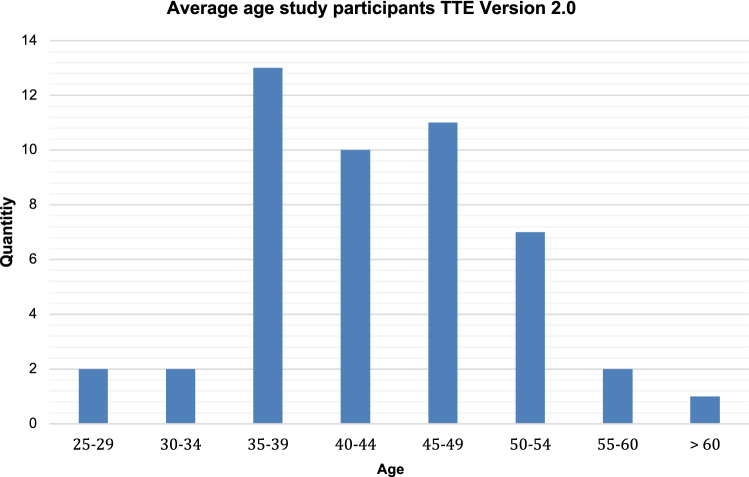
Average age of the TDSC course participants of the game version 2.0 with application on the *x*-axis: age and on the y-axis number of participants. 92% (*n* = 44 of 48) of the course participants were over 34 years old, the most represented group was the 35–39-year-old group with *n* = 13, i.e., 27%, the minimum age was in the 25–29-year-old group with two course participants, the maximum age was defined by one course participant who stated that he was over 60 years old

**Fig. 3:**
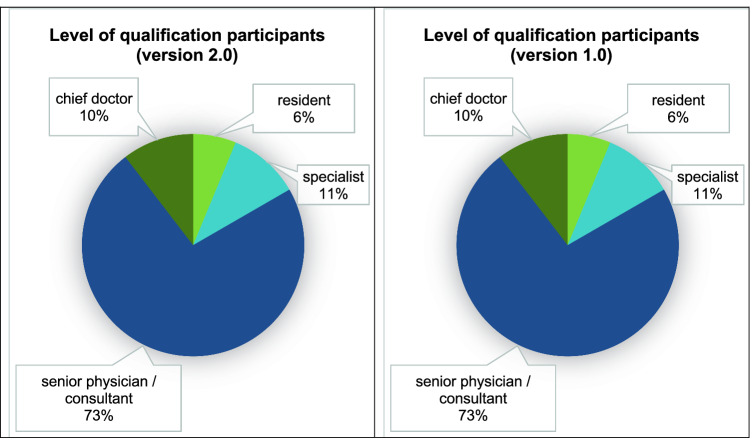
Training level of the participants, left version 2.0, right version 1.0. The percentages of the respective training level of the participants are shown. In version 2.0, the proportion of residents was 6%, of specialists 11%, of senior physicians 73%, of chief physicians 10%. In version 1.0, the percentage of residents was 8%, of specialists 26%, of attendings 53%, of chiefs 13%

**Fig. 4:**
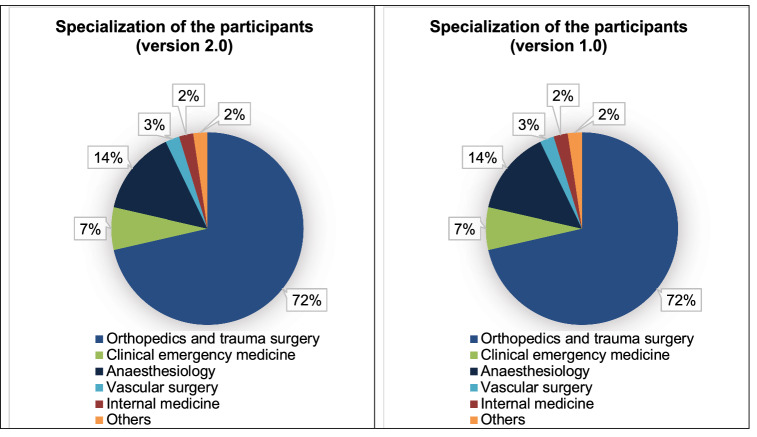
Composition of specialties, left for exercise 2.0, right for version 1.0. For version 2.0, 72% of participants were from orthopedics and trauma surgery, 14% from anesthesiology, 7% additional training in clinical emergency medicine, 3% vascular surgery, 2% internal medicine, 2% other specialties. For version 1.0, the proportion of the specialty of trauma surgery and orthopedics was 39%, anesthesiology 41%, other specialties with *n* = 6 were composed of ENT *n* = 1, neurosurgery *n* = 1, visceral surgery *n* = 1 vascular surgery *n* = 1, internal medicine *n* = 1, health economics *n* = 1

A further analysis of the participants in terms of bibliographic data, their field of study and experience horizon was performed at the beginning of the courses, Figs. 2, 3 and 4 show the results.

### Sighting frequency and hit accuracy of the triage categories

**Fig. 5:**
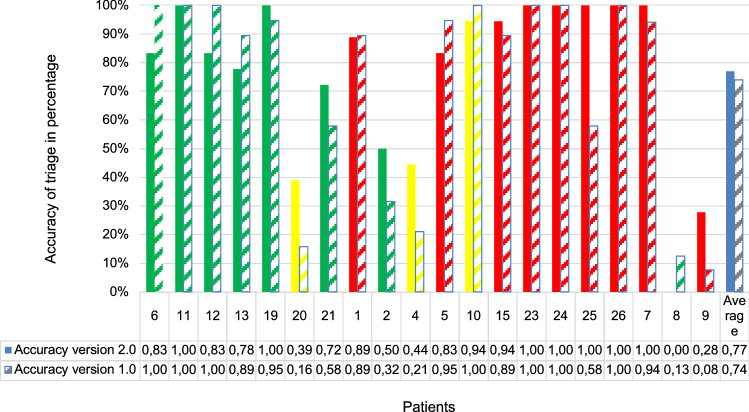
Hit accuracy of the triage categories in comparison of the table-top exercise version 2.0–1.0: *x*-axis: patients as they were imported in chronological order within the scenario, *y*-axis: hit accuracy of the triage category in percent, the mean value is shown on the far right. Full color bars (green, red, yellow, blue) represent the results for version 2.0, saded bars (green, red, yellow, blue) for version 1.0

The main outcome measure "allocation of patients for the correct triage category" was evaluated as indicated with patients *n* = 360 in version 2.0 and *n* = 369 in version 1.0 (Fig. 5). As mentioned above 11 patient cards had not yet been viewed in the evaluation of version 1.0 due to a predefined game time limit after 90 min and were, therefore, not consecutively included in the game evaluation.

In the version 2.0, 44.4% of the patient cards were red sighted; in version 1.0, the percentage of red-sighted patient cards was 43.4%. The percentage of patient cards sighted yellow in version 2.0 was 23.6%; in version 1.0, 21.7%. The proportion of green sighted patient cards in version 2.0 was 31.9%; in version 1.0, 35.0%. With regard to the correctness of the category assignment, the following information can be provided:

Red patients *n* = 9 (patient 1, 5, 7, 9, 15, 23, 24, 25) were seen 88.22% correct (minimum 28%, maximum 100%) in version 2.0, in version 1.0 81.41% (minimum 7,7%, maximum 100%).

Yellow triaged patients *n* = 3 (patient 4, 10, 20) were sighted 59.0% correctly (minimum 39%, maximum 100%) in version 2.0, and 45.67% correctly (minimum 16%, maximum 100%) in version 1.0.

Green triaged patients *n* = 8 (patient 2, 6, 8, 11, 12, 13, 19, 21) were correctly sighted with 70.75% (minimum 0%, maximum 100%) in version 2.0, and with 73.41% (minimum 13%, maximum 100%) in version 1.0.

Overall, the average accuracy of the sighting categories was 76.94% in version 2.0 and 74.25% in version 1.0 (Fig. 5).

### Evaluation of the location of patients at the end of the game

**Fig. 6:**
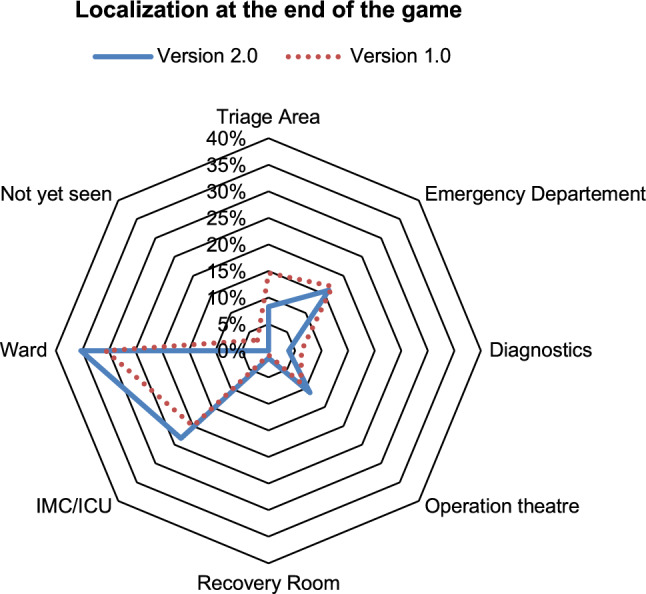
Location of patient cards relative in percent depending on the respective total number of patient cards (*n* = 369 for version 1.0, *n* = 360 for version 2.0) at the end of the game. The picture is very similar between exercise version 2.0 and 1.0

Figure 6 shows the location of the patients at the end of the game ("Where is which patient located at the end of the game?"). For the total of 729 patients played, a very similar and homogeneous picture between version 2.0 and 1.0 emerges. Thus, for the individual areas can be cited:

- Triage area: version 2.0 8% vs. version 1.0 15%.
- Emergency department Treatment area "red" and "yellow": version 2.0 16% vs. version 1.0 17%.
- Diagnostics: version 2.0 4% vs. version 1.0 6%.
- Operation theater: version 2.0 11% vs. version 1.0 9%.
- Recovery room: Version 2.0 1% vs. Version 1.0 1%
- Intermediate Care Unit (IMC)/Intensive Care Unit (ICU): version 2.0 23% vs. Version 1.0 20%.
- Ward: Version 2.0 35% vs. Version 1.0 31%.
- Not yet seen: Version 2.0 0% vs. version 1.0 3%.

### Evaluation of the diagnostics performed at the end of the game

**Fig. 7:**
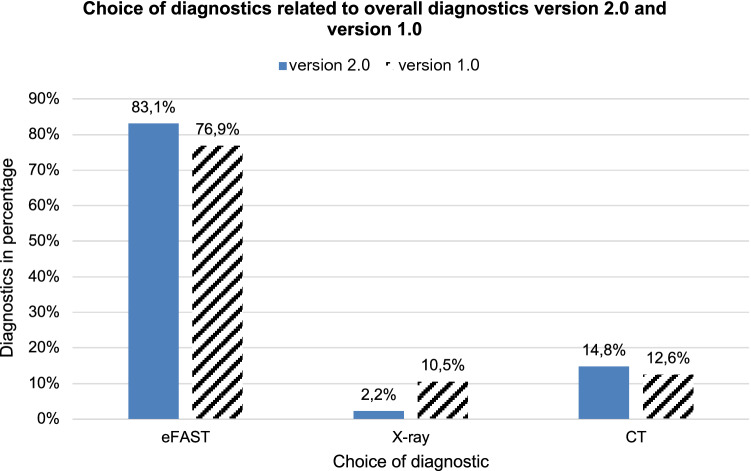
Selected diagnostics of patients in percent. In the game version 2.0 *n* total = 183. In the game version 1.0 *n* total = 247. *x*-axis: diagnostics, *y*-axis percentage frequency

The selected diagnostics of the patients were evaluated as a percentage. For version 2.0, the total number of all diagnostic measures performed was *n* = 183. For version 1.0, the total number of diagnostic measures performed was *n* = 247. The proportion of diagnostic measures for eFAST examinations in version 2.0 was 83.1% and 76.9% in version 1.0. X-ray examinations were 2.2% for version 2.0 and 10.5% for version 1.0. Furthermore, CT examination was performed in 14.8% (version 2.0) vs. 12.6% (version 1.0). (Fig. 7).

### Evaluation of the defined therapy decisions at the end of the game

**Fig. 8:**
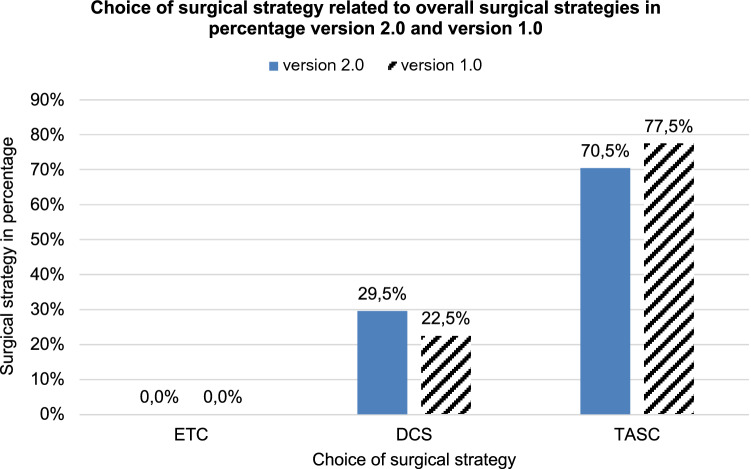
Selected form of therapy in the patient charts relative. *x*-axis therapy type, *y*-axis therapy in percent. Game version 2.0: DCS *n* = 31, TASC *n* = 74; game version 1.0: DCS *n* = 18, TASC *n* = 62

Furthermore, an evaluation of the selected form of therapy was carried out for the patients as a percentage. In version 2.0, the total number of all therapeutic procedeures carried out was *n* = 105, in the old game *n* = 80; Fig. 8 shows the more extensive distribution here.

- In the game version 2.0 as well as in the version 1.0 no therapy is carried out according to the early total care (ETC)—principle.
- According to the concept of damage control surgery (DCS) 29.5% of patients were treated in version 2.0 and 22.5% in version 1.0.
- Tactical abbreviated surgical care (TASC) was chosen for 70.5% of patients in version 2.0 and 77.5% in version 1.0.

### Evaluation of the TDSC course instructors

A further prospective monocentric evaluation of the TDSC^®^ course instructors was carried out, within the courses with the table-top exercise of version 2.0. The task of the instructors within the course is to guide and supervise the exercise, so that a goal-oriented and rule-compliant process is ensured. Since the questionnaire is about a comparison between the new and the old exercise, only instructors who had already instructed version 1.0 were selected to ensure an adequate significance with regard to the comparability between the table-top exercise version 2.0 vs. version 1.0. In addition, the evaluation was not carried out immediately with the introduction of the new game, but preliminary courses with version 2.0 were held first, so that the instructors were given the opportunity to familiarize themselves with the new exercise. A total of 16 course instructors participated in the evaluation, 16 of whom were male. Fourteen of the 16 participants are active in surgery, and two are active in anesthesiology. The median age of the course instructors was most pronounced in the 41–50 age group with a number of *n* = 9, 4 could be assigned to the 31–40 age group, and 3 to the 51–60 age group. Five of the instructors worked in the clinical routine in the position of a chief physician or in a leading position, ten as a senior physician, one as a specialist, whereby all but one instructor came from a level-I trauma center.

Regarding the level of experience in working as an instructor, it was shown that 15 instructors had been course instructors for at least 12 months, one person stated that he had been working for less than 13 months, and half of the participants had even been actively involved in the courses for more than 36 months.

On median, instructors had facilitated 7 or more courses.

Instructors rated the learning effect on terror preparedness through the simulation in school grades (1 = very good, 6 = unsatisfactory). Six of the instructors gave a grade of 1, and ten gave a grade of 2.

The feel of the table-top exercise was consistently rated as significantly improved for version 2.0, 13 of the instructors also indicated a greater learning effect, three rated this as the same compared to version 1.0.

Eight instructors found it easier to explain version 2.0 compared to version 1.0, 7 considered this to be the same, only one instructor stated that it was not easier for him.

In the sense of an overall, the instructors rated the version 2.0 of the table-top exercise (in school grades 1–6) with a mean of 1.53 with a median of 1.50 and a standard deviation s = 0.44 (in school grades (1 = very good, 6 = insufficient). The old game was rated with a mean of 2.3188 at a median of 2.15 with a standard deviation *s* = 0.66.

## Discussion

The data collected in this study suggest an evaluation of the table-top exercise in the Terror and Disaster Surgical Care (TDSC^®^) course as an educational component for more advanced terror preparedness among clinical decision-makers as purposefully possible. This will enable a differentiated description, analysis and discussion.

With the help of this work, it should be examined whether a table-top exercise is suitable for preparing clinical decision-makers for in-hospital crisis situations, especially in terror-associated large-scale damage facilities. This can be confirmed on the basis of the present results and is in line with previous experience from the literature. The effectiveness of simulation games can also be found elsewhere in the literature [11, 12].

Looking at the main target variable for the hit accuracy of sighting categories in a game round that was repeatedly run in the same way in a total of six courses, it could be shown that there was a homogeneous decision-making behavior. Stably the patients played in the context of the scenario were assigned to the categories red, yellow or green (SK I, II or III), whereby also no difference between the table-top exercise of the version 1.0 to version 2.0 was to be described.

The same applies to the secondary outcome measures that were also examined. Both for the location of the patients at the end of the game round and the diagnostics and conceptual therapy decision carried out up to that point, the results for the 6 courses with nearly 100 participants and over 720 recorded patient cards were very homogeneous.

It may be deduced that the training format of the course with the educational knowledge transfer, the exemplary case presentations and their interactive discussions could provide the participants with appropriate basics. Basics in the sense of the special features of such large-scale incidents, the clinical consequences to be derived from them with correspondingly different injury patterns (keyword: penetrating injuries, BLAST injuries) and the resulting very threatening secondary conditions of the immediately life-threatening hemorrhage, septic secondary conditions, especially in the body cavities and the five different entities of the BLAST injury. Thus, the participants were able to make correct and consistent decisions for the emerging patients, to apply the trained basic understanding also in strategic–tactical terms and also to apply newly learned and previously unknown therapeutic concepts, such as Tactical Abbreviated Surgical Care.

This could also be proven by the evaluation of the course instructors, who could confirm this impression from the point of view and function of a kind of "referee" during the respective table-top exercises. In particular, the question about the impression of the learning process should be mentioned, as well as the improvement and optimization of the table-top exercise from version 1.0 to version 2.0.

As previously mentioned, the patients in the simulation games were triaged using the Berlin screening algorithm [13]. Patient screening plays a key role in the successful management of mass casualty incidents and is used for the correct allocation of resources. The analysis of unannounced disaster control exercises in Berlin hospitals showed problems with the correct classification of patients into the triage categories with a relevant over- and under-triage. Therefore, a hospital screening algorithm was developed and introduced in Berlin in 2015 called the Berlin Sichtungsalgorithmus.To check the effects and validate the triage algorithm Kleber et al. conducted a prospective study in 2020 [13]. In summary, the Berlin screening algorithm was highly effective with a specificity and sensitivity of 97/75% for SKI and 86/67% for SKII, 85/88% for SKIII patients. The question here was when there was a higher hit accuracy of the sighting categories. A significantly better sighting result of 80% with the Berlin sighting algorithm compared to 63% without a sighting algorithm could be determined. The screening result with 80% of the study by Kleber et al. from 2020 corresponds approximately to the sighting result/ hit accuracy of 76.94% of the results of the version 2.0 of our table-top exercise. Consequently, a high hit accuracy of the screening categories in the TDSC^®^ simulation game can be derived, since here the screening was based on playing cards, whereas in the previously mentioned study design this was based on real injured actors. It can be suspected that the study participants find it even easier to carry out a sighting on real wounded people than on playing cards. The fact that the accuracy of hits in the evaluated table-top exercise is almost the same as in the study described above shows all the more the high quality of the simulation game. A study by Ingrassia et al. from 2010 presented the hit accuracy of the triage using the Simple Triage and Rapid Treatment (START) algorithm. Here, the probands viewed 127 injured people (medical students) from the University of Piedmont in Novara, Italy. A hit accuracy of 81% was shown, with 100% of the green, 61% of the yellow and 67% of the red patients being correctly identified. The overall hit accuracy of the START screening algorithm used in this study is with 81% vs 76,94% higher than that of the Berlin screening algorithm used for our study. However, this can be explained by the fact that the green patients were viewed correctly within 100% which raises the overall average, whereas yellow and red patients were often not recognized correctly. As described the hit accuracy in our simulation game of the green was 70.75% for version 2.0 and 73.41% for version 1.0. Yellow patients were correctly identified 59% in version 2.0 and 45.67% in version 1.0. The proportion of the acutely life-threatening red screening category is impressive: in version 2.0 88.22% of the patients were recognized as really red, in version 1.0 81.41% of the patients. Since the red patients in particular have to be recognized correctly sighted in a TerrorMANV due to their vital threat, there is a much higher hit accuracy of 88.22% version 2.0 compared to the START sighting algorithm compared to the hit accuracy there for the red sighting category of only 67%. This proves the superiority of the Berlin sighting algorithm used in the TDSC^®^ course.

Intensive training and continuing education of decision-makers within the hospital for this area will continue to play an important role [14]. In the future, the focus will not have to be on terror-associated major emergencies, for example. With regard to Germany, events such as the mass casualty incident from the air show in Rammstein in 1988 as well as the accident of the ICE express train near Eschede in 1998 have shown that we have also been challenged in the past with major challenges for the medical care system due to a large number of severely and most severely injured patients. Natural disasters also accompany us in this respect, and here, for example, the flood of the Ahr in 2021, which was also considered for Germany, clearly brought the necessities and problems to light. Each entity of a major damage event has its own challenges, to which it is necessary to adapt individually. However, it is all the more important to be prepared, because only then is it possible to fall back on concepts that exist in thought and hopefully concepts that exist and have been practiced within the clinic [2].

In this context, we are confronted with the challenge of imparting appropriate content and knowledge, and ideally also practicing this, in the area of tension between the requirements of normal clinical everyday life and also increasingly scarce personnel and financial resources. Experience shows that a complete implementation through real-life and real-time as well as full-scale exercises in everyday life is not possible. It is therefore necessary to implement and expand other training modules [15]. In this respect, the TDSC^®^ course is exemplary in its basic approach, similar to the MRMI - course with a different focus [4]. With the offered course structure and in particular the implementation of the table-top excercise a corresponding target achievement may be assumed. This is especially true since the topic of board games is gaining increasing educational recognition.

Further developments must follow, however, to bring about a constant optimization. Digitalization is certainly a major focus here and is already being used in the first steps for the implementation of the TDSC^®^ course, especially the table-top exercise. This should also ensure acceptance by future generations of young colleagues and a continued stable interest in the use of such techniques.

Achatz et al. were able to show in the past already nicely that the simulation game is perceived as purposeful and successful [10].

## Conclusion

Finally, it can be summarized that the simulation is suitable for the training project to prepare clinical decision-makers for large-scale incidents such as a terrorMASCAL. The most recently introduced changes, adaptations and improvements in the TDSC^®^ courses have led to an even higher level of acceptance with a consistently high level of training success. Further development must be driven forward, particularly in view of increasing digitization, and a corresponding study evaluation must accompany these processes.

## References

[1] Achatz G, Bieler D, Franke A (2020). Terror preparedness as a service of general interest: the Terror and Disaster Surgical Care (TDSC^®^)-course. Eur J Trauma Emerg Surg.

[2] Hoth P, Bieler D, Friemert B et al. Sicherheitsaspekte und Vorbereitung zur Notfallvorsorge und Gefahrenabwehr in Kliniken bei MANV/TerrorMANV : Ausblick auf zukünftige Herausforderungen anhand von Umfrageergebnissen zur 3. Notfallkonferenz der DGU, *Der Unfallchirurg*, 2021. 10.1007/s00113-021-01046-y PMC9256572 34338840

[3] Marung H, Birkholz T, Dittmar MS (2014). Der Leitende Notarzt – etablierte Konzepte und neue Anforderungen. Notfallmedizin up2date.

[4] Montán KL, Örtenwall P, Lennquist S (2015). Assessment of the accuracy of the Medical Response to Major Incidents (MRMI) course for interactive training of the response to major incidents and disasters. Am J Disaster Med.

[5] Gauthier A, Kato PM, Bul KCM (2019). Board games for health: a systematic literature review and meta-analysis. Games Health J.

[6] Skryabina E, Reedy G, Amlôt R (2017). What is the value of health emergency preparedness exercises? A scoping review study. Int J Disaster Risk Reduct.

[7] Prengel P. 012-019l_S3_Polytrauma_Schwerverletzten-Behandlung_2017–08.

[8] Deutscher Ärzteverlag GmbH, Redaktion Deutsches Ärzteblatt, “Versorgung von Terroropfern: Neue Ausbildungsformate,”. 2022. https://www.aerzteblatt.de/archiv/183000/Versorgung-von-Terroropfern-Neue-Ausbildungsformate.

[9] Achatz G, Friemert B, Trentzsch H (2020). Terror and disaster surgical care: training experienced trauma surgeons in decision making for a MASCAL situation with a tabletop simulation game. Eur J Trauma Emerg Surg.

[10] Trentzsch H. Table top exercise f. Instruktoren_V3.5_Stand 10.07.2020.

[11] Gilsdorf E. Board games are back, and Boston’s a player. A Golden Age of tabletop games, from nerdy to mainstream, is afoot. The Boston Globe Magazine; 2014.

[12] Noda S, Shirotsuki K, Nakao M (2019). The effectiveness of intervention with board games: a systematic review. BioPsychoSoc Med.

[13] Kleber C, Solarek A, Cwojdzinski D (2020). Der Berliner Krankenhaus-Sichtungsalgorithmus für den Massenanfall von Verletzten : Entwicklung, Implementierung und Einfluss auf übungsbasierte Sichtungsergebnisse. Unfallchirurg.

[14] Nishisaki A, Keren R, Nadkarni V (2007). Does simulation improve patient safety? Self-efficacy, competence, operational performance, and patient safety. Anesthesiol Clin.

[15] Nicholson S (2011). Making gameplay matter: designing modern educational tabletop games. Knowl Quest.

